# ZnCl_2_ sustains the adriamycin-induced cell death inhibited by high glucose

**DOI:** 10.1038/cddis.2016.178

**Published:** 2016-06-30

**Authors:** A Garufi, D Trisciuoglio, M Cirone, G D'Orazi

**Affiliations:** 1 Department of Research, Advanced Diagnostics, and Technological Innovation, Regina Elena National Cancer Institute, Rome, Italy; 2 Department of Medical, Oral and Biotechnological Sciences, Tumor Biology Section, University ‘G. d'Annunzio', Chieti, Italy; 3 Department of Experimental Medicine, Pasteur-Fondazione Cenci Bolognetti Institute, Sapienza University, Rome, Italy

## Abstract

Hyperglycemia, the condition of high blood glucose, is typical of diabetes and obesity and represents a significant clinical problem. The relationship between hyperglycemia and cancer risk has been established by several studies. Moreover, hyperglycemia has been shown to reduce cancer cell response to therapies, conferring resistance to drug-induced cell death. Therefore, counteracting the negative effects of hyperglycemia may positively improve the cancer cell death induced by chemotherapies. Recent studies showed that zinc supplementation may have beneficial effects on glycemic control. Here we aimed at evaluating whether ZnCl_2_ could counteract the high-glucose (HG) effects and consequently restore the drug-induced cancer cell death. At the molecular level we found that the HG-induced expression of genes known to be involved in chemoresistance (such as HIF-1*α*, GLUT1, and HK2 glycolytic genes, as well as NF-*κ*B activity) was reduced by ZnCl_2_ treatment. In agreement, the adryamicin (ADR)-induced apoptotic cancer cell death was significantly impaired by HG and efficiently re-established by ZnCl_2_ cotreatment. Mechanistically, the ADR-induced c-Jun N-terminal kinase/stress-activated protein kinase (JNK/SAPK) phosphorylation, inhibited by HG, was efficiently restored by ZnCl_2_. The JNK involvement in apoptotic cell death was assessed by the use of JNK dominant-negative expression vector that indeed impaired the ZnCl_2_ ability to restore drug-induced cell death in HG condition. Altogether, these findings indicate that ZnCl_2_ supplementation efficiently restored the drug-induced cancer cell death, inhibited by HG, by both sustaining JNK activation and counteracting the glycolytic pathway.

The complex interplay between cancer cells and the surrounding microenvironment may both play a role in cancer progression and negatively affect cancer treatment. Hyperglycemia is a condition in which an excessive amount of glucose circulates in the blood that develops when the body has too little insulin or when the body cannot use insulin properly.^1^ The most common condition responsible for hyperglycemia is diabetes mellitus, but other medical conditions can cause hyperglycemia, including obesity, pancreatitis, chronic stress, and cancer.^2, 3, 4, 5, 6, 7^ It appears that hyperglycemia may contribute to a more malignant cancer phenotype and that might inhibit tumor response to therapies, conferring resistance to chemotherapy-induced cell death.^8, 9, 10, 11, 12^ A successful anticancer therapy is achieved when cancer cells undergo apoptosis.^13^ Therefore, intrinsic or acquired deregulation in apoptotic cell death machinery becomes a determinant factor for cancer resistance to therapies.^14^ For that reason, resistance to apoptosis is one of the distinctive hallmarks of cancer.^15^ Adriamycin (ADR), an anthracycline antibiotic, is one of the most potent drugs used for therapy of malignancies, including colon cancer, leading to cell death through several mechanisms,^16^ although acquired drug resistance is a major limiting factor in its clinical applications.^17^ One potential apoptotic signaling pathway known to be activated by ADR is the c-Jun N-terminal kinase/stress-activated protein kinase (JNK/SAPK) pathway.^18, 19^

It is well established that cancer cells are addicted to glucose and very sensitive to glucose concentration changes;^20^ indeed, glucose is the primary driving force for the growth of tumor cells, whereas glucose deprivation induces growth inhibition and cancer cell death.^21^ A distinct trait of the cancer metabolism is the unscheduled activation of glycolytic enzymes in normoxic conditions, indicated by increased cellular glucose uptake, hyperglycolysis, and lactate production for energy production in an oxygen-independent manner.^22^ Thus, targeting glycolysis remains attractive for therapeutic intervention.^23^ The increased glucose transport in cancer cells has been attributed primarily to the upregulation of glucose transporter isoform 1 (GLUT1), one of the main controlling steps of the glycolytic flux in cells.^24^ GLUT1 is often overexpressed in several solid tumors and is associated with tumor progression and chemoresistance.^25, 26, 27^ GLUT1 as well as glycolytic enzyme hexokinase 2 (HK2) are upregulated by the hypoxia-inducible factor-1*α* (HIF-1*α*),^28^ a transcriptional factor often activated in solid tumors and whose function is involved in tumor progression, metabolic reprogramming, and chemoresistance.^29^ High-glucose (HG) condition also predisposes to a proinflammatory phenotype through activation of nuclear factor (NF)-*κ*B transcription factor.^30^ The cross-talk between inflammatory and tumor cells has been demonstrated to be pivotal for cancer development^15^ and the transcription factor NF-*κ*B represents the main molecular link between inflammation and cancer.^31^ Of note, NF-*κ*B has been shown to mediate antiapoptotic activities by blocking the activation of JNK.^32^ On the basis of these evidences, counteracting the effects of hyperglycemia and the glycolytic pathway may have important therapeutic implications in cancer patients. In this regard, we previously demonstrated that zinc chloride (ZnCl_2_) supplementation in cancer cells can inhibit HIF-1*α* activity,^33^ counteracting the hypoxia-induced chemoresistance^34, 35^ and the proinflammatory phenotype. Based on the above background, in the present study we aimed at evaluating whether ZnCl_2_ supplementation could inhibit the glycolytic pathway induced by HG condition and consequently enhance the cytotoxic activity of antineoplastic agent *in vitro* in cancer cells.

## Results

### The HG-induced expression of hypoxic/glycolytic genes is counteracted by ZnCl_2_ cotreatment

**Figure 1:**
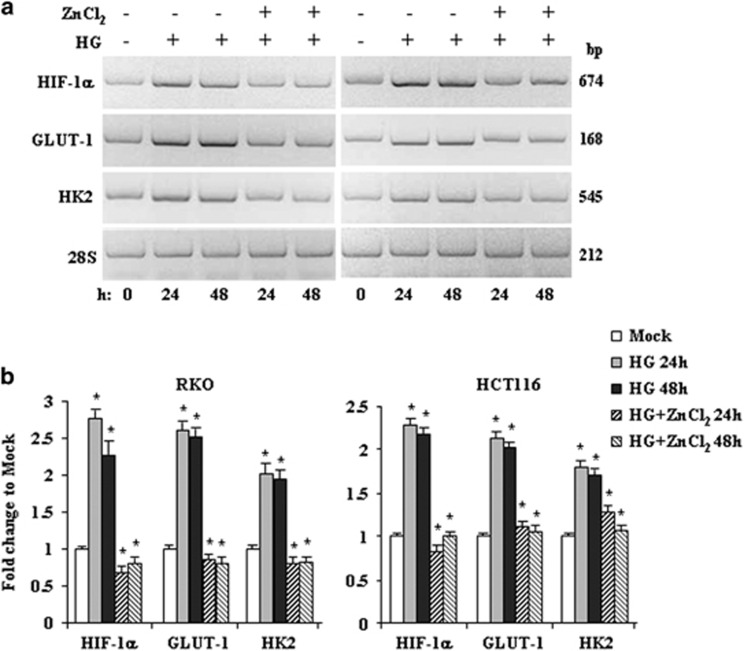
The HG-induced gene expression is counteracted by ZnCl_2_ cotreatment. (**a**) RKO and HCT116 cells were grown in HG condition for 24 and 48 h with or without ZnCl_2_ (100 *μ*M) before being assayed for semiquantitative RT-PCR of the indicated genes. The 28S was used as a control for efficiency of RNA extraction and transcription. The genes' size is indicated in base pair (bp). (**b**) Densitometric analysis for quantification of the bands as 28S/gene ratio. Data are presented as mean±S.E.M. of *n*=4 (RT-PCR) (one-way ANOVA plus Bonferroni test, *P<0.01 HG 24 h *versus* Mock, HG 48 h *versus* Mock, HG+ZnCl_2_ 24 h *versus* HG 24 h, HG+ZnCl_2_ 48 h *versus* HG 48 h)

To evaluate whether the HG-induced gene expression could be modified by concomitant ZnCl_2_ treatment we performed *in vitro* analyses of mRNA levels by reverse transcription-PCR (RT-PCR). Colon cancer RKO and HCH116 cells were cultured in HG (4.5 g/l D-glucose, considered HG condition)^36^ for 24 and 48 h, alone or in combination with ZnCl_2_. The results show that the HG culture condition upregulated HIF-1*α*, GLUT1, and HK2 glycolytic enzyme and that such mRNA overexpression was markedly inhibited by ZnCl_2_ cotreatment (Figure 1a), as also evidenced by densitometric analyses (Figure 1b).

### ADR-induced cell death was impaired by HG but sustained by ZnCl_2_ cotreatment

**Figure 2:**
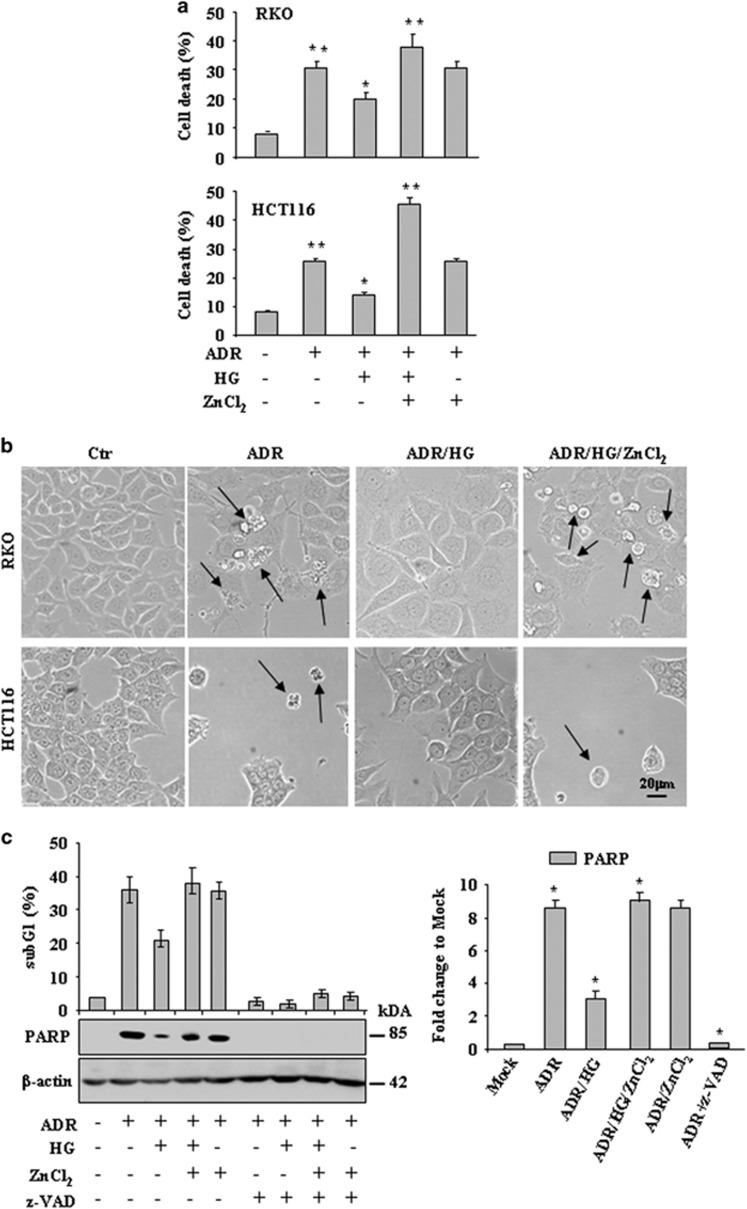
The ADR-induced cell death is reduced by HG and re-established by ZnCl_2_ cotreatment. (**a**) RKO and HCT116 cells (2 × 10^5^) were plated at subconfluence in culture media containing 10% FBS and 1 g/l D-glucose. The day after, medium was changed with medium containing 2% FBS with either 1 g/l D-glucose (low glucose) or 4.5 g/l D-glucose (HG) for 24 h before adding chemotherapeutic drugs ADR (2 *μ*g/ml) with or without ZnCl_2_ (100 *μ*M). After 24 h, the percentage of dead cells was scored by Trypan blue exclusion. Data are presented as mean±S.E.M (*n*=6) (one-way ANOVA plus Bonferroni test, **P<0.001 ADR *versus* Mock and ADR/HG/ZnCl_2_ *versus* ADR/HG, *P<0.01 ADR/HG *versus* ADR). (**b**) Live images of cells analyzed in (**a**) were taken before lysing cells. Black arrows indicate dead cells. (**c**) RKO cells were treated with ADR (2 *μ*g/ml) in low and high glucose condition with or without ZnCl_2_ (100 *μ*M) for 24 h; the irreversible caspase inhibitor z-VADfmk was used at 40 mM for 16 h. After treatments, cells were in part fixed and stained with PI for sub-G1 evaluation (upper panel) or lysed and analyzed by western immunoblotting to assess PARP cleavage (lower panel) and relative quantification of PARP cleavage/*β*-actin ratio (right panel). Anti-*β*-actin was used as protein loading control. The predicted molecular weight is indicated (kDa). For sub-G1 analysis, data are presented as mean±S.D. In the right panel data are presented as mean±S.E.M. (*n*=6) (one-way ANOVA plus Bonferroni test, *P<0.001 ADR *versus* Mock, ADR/HG *versus* ADR, ADR/HG/ZnCl_2_ *versus* ADR/HG, ADR+z-VAD *versus* ADR)

In light of the finding that ZnCl_2_ might downregulate some HG-induced glycolytic genes that have been shown to protect cancer cells from cell death induced by chemotherapy,^8, 10, 29, 36^ we aimed at evaluating whether the antiapoptotic effect of HG could be inhibited by ZnCl_2_ cotreatment. To this aim, RKO and HCT116 colon cancer cells, cultured in low- or high-glucose condition, were treated with of the antineoplastic agent ADR with or without ZnCl_2_ cotreatment. Cell viability assay showed that the ADR-induced cell death, impaired by HG condition, was significantly abrogated by ZnCl_2_ cotreatment (Figure 2a), as also evidenced microscopically (Figure 2b). Next, cell death was also examined by fluorescence-activated cell sorting (FACS) analysis of propidium iodide (PI)-stained sub-G1 cell population. As shown in Figure 2c (upper panel), the ADR-induced cell death was significantly reduced by HG and efficiently re-established by ZnCl_2_ cotreatment. In agreement, western blot analysis showed that the ADR-induced poly(ADP-ribose) polymerase (PARP) cleavage (indicative of apoptotic cell death)^37^ was strongly reduced by HG and markedly restored by ZnCl_2_ cotreatment (Figure 2c, lower panel), as also assessed by densitometric analysis (Figure 2c, right panel). The use of the irreversible, cell-permeable, pancaspase inhibitor z-VAD-fmk confirmed the apoptotic nature of ADR-induced cell death (Figure 2c). Altogether, these data indicate that the ADR apoptotic effect, reduced by HG, could be efficiently re-established by ZnCl_2_ cotreatment.

### ZnCl_2_, similar to GLUT1 downregulation or glycolysis inhibition by 2-DG, re-established the ADR-induced cell death inhibited by HG

**Figure 3:**
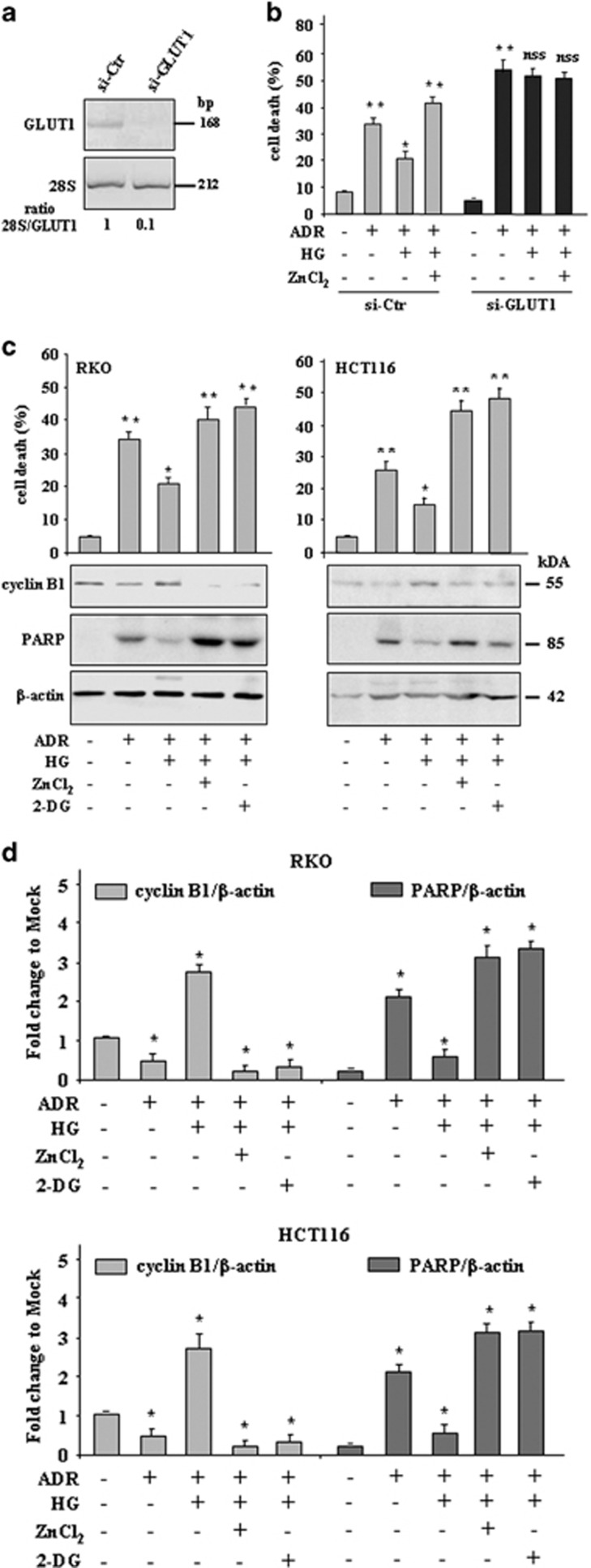
ZnCl_2_ supplementation, similar to GLUT1 downregulation or glycolysis inhibition by 2-DG, re-established the ADR-induced cell death reduced by HG. (**a**) RKO cells were transfected with siRNA for GLUT1 (si-GLUT1) or with control siRNA (si-Ctr). At 36 h after transfection, cells were analyzed for GLUT1 expression by RT-PCR. The gene's size is indicated in base pair (bp). The 28S/GLUT1 ratio is indicated. (**b**) Cells interfered as in (**a**) were treated with ADR (2 *μ*g/ml) in low and high glucose condition with or without ZnCl_2_ (100 *μ*M) for 24 h, before being assayed for cell viability by Trypan blue exclusion. Data are presented as mean±S.E.M and the results of one-way ANOVA plus Bonferroni test are as in Figure 2a for si-Ctr samples; **P<0.001 ADR (si-GLUT1) *versus* ADR (si-Ctr), not statistically significant (NSS) ADR/HG (si-GLUT1) *versus* ADR (si-GLUT1) and ADR/HG/ZnCl_2_ (si-GLUT1) *versus* ADR/HG (si-GLUT1). (**c**) RKO and HCT116 cells were treated with ADR 2 (*μ*g/ml) in low and high glucose condition with or without ZnCl_2_ (100 *μ*M) for 24 h; the glycolytic inhibitor 2-DG was used at 12 mM for 24 h. After treatments, cells were assayed for cell viability by Trypan blue exclusion (upper panel). Data are presented as mean±S.E.M. (*n*=6) (one-way ANOVA plus Bonferroni test, **P<0.001 ADR *versus* Mock, ADR/HG/ZnCl_2_ *versus* ADR/HG, ADR/HG/2-DG *versus* ADR/HG, *P<0.01 ADR/HG *versus* ADR). Then, cells were lysed and analyzed by western immunoblotting to assess PARP cleavage and cyclin B1 levels (lower panel). Anti-*β*-actin was used as protein loading control. The predicted molecular weight is indicated (kDa). (**d**) Densitometric analysis was applied to quantify PARP cleavage/*β*-actin ratio and cyclin B1/β-actin ratio. Data are presented as mean±S.E.M. (one-way ANOVA plus Bonferroni test, *P<0.001 ADR *versus* Mock, ADR/HG *versus* ADR, ADR/HG/ZnCl_2_ *versus* ADR/HG, ADR/HG/2-DG *versus* ADR/HG)

GLUT is one of the main controlling steps of the glycolytic flux in cells.^24^ GLUT1 overexpression is associated with tumor progression and chemoresistance in several solid tumor cells including colon.^25, 26^ To corroborate the finding that targeting the glucose transport pathway could restore tumor response to ADR, RKO cells were transfected with GLUT1 small interference RNA (siRNA) to silence GLUT1 expression. The efficiency of RNA interference was monitored at the RNA level by RT-PCR (Figure 3a). Inhibition of GLUT1 by RNA interference increased the cell sensitivity to ADR cytotoxicity (Figure 3b, compare ADR (si-GLUT-1) with ADR (si-Ctr)) that could not be inhibited by HG treatment (Figure 3b, compare ADR/HG *versus* ADR (si-GLUT-1) with ADR/HG *versus* ADR (si-Ctr)), and was not further enhanced by ZnCl_2_ cotreatment (Figure 3b, compare ADR/HG/ZnCl_2_ *versus* ADR/HG (si-GLUT-1) with ADR/HG/ZnCl_2_ *versus* ADR/HG (si-Ctr)). These findings confirm the link between GLUT1 overexpression and acquired chemoresistance and indicate that GLUT1 is a target of ZnCl_2_.

To further assess whether ZnCl_2_ could target the glycolytic pathway, thus improving drug cytotoxicity, we used the glycolytic inhibitor 2-deoxy-D-glucose (2-DG),^38, 39^ in comparison with ZnCl_2_ treatment. Treatment with 2-DG markedly restored the ADR-induced cell death inhibited by HG (Figure 3c); interestingly, ZnCl_2_ showed similar effect to that of 2-DG (Figure 3c), suggesting that it could indeed target the glycolytic pathway. At the molecular level, we analyzed both the cleavage of the apoptotic marker PARP^37^ and the expression levels of cyclin B1 that in mammalian cells plays a role in the cell cycle progression through mitosis.^40^ As shown in Figure 3c (lower panels), in the presence of apoptotic cell death (i.e., samples: ADR; ADR/HG/ZnCl_2_; ADR/HG/2-DG) we found efficient PARP cleavage and, in an opposite way, marked reduction of cyclin B1 levels; moreover, when ADR-induced cell death was reduced by HG, PARP cleavage was also strongly reduced whereas cyclin B1 expression was increased, in both cell lines (Figure 3c, lower panels), as also assessed by densitometric analysis (Figure 3d). Altogether, these findings show that ZnCl_2_, similar to GLUT1 downregulation or glycolysis inhibition by 2-DG, re-established the ADR-induced cell death inhibited by HG. These data suggest that ZnCl_2_ might be a valuable glycolytic inhibitor to use in anticancer combination therapy to sustain drug cytotoxicity.

### ZnCl_2_ cotreatment sustains the ADR-induced JNK phosphorylation inhibited by HG condition

**Figure 4:**
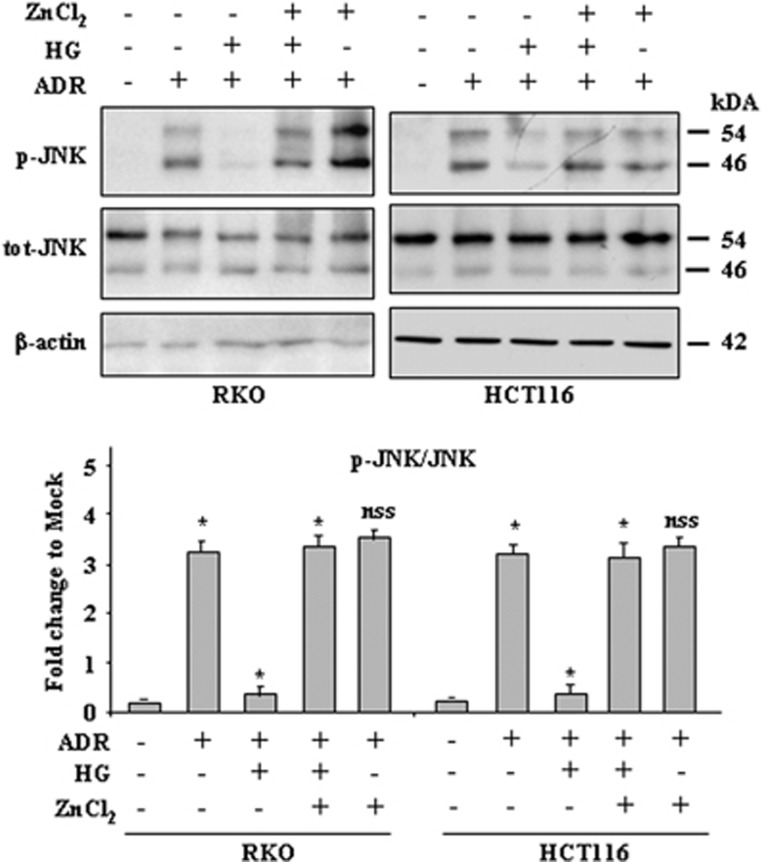
ZnCl_2_ cotreatment restores the ADR-induced JNK phosphorylation inhibited by HG condition. Western blotting (upper panel) and relative quantification (lower panel) of p-JNK/tot-JNK ratio in RKO and HCT116 cells treated with ADR (2 *μ*g/ml) in low and high glucose condition with or without ZnCl_2_ (100 *μ*M) for 24 h. Anti-*β*-actin was used as protein loading control. The predicted molecular weight is indicated (kDa). Data of relative quantification of p-JNK/tot-JNK ratio (lower panel) are presented as mean±S.E.M. (*n*=6) (one-way ANOVA plus Bonferroni test, *P<0.001 ADR *versus* Mock, ADR/HG *versus* ADR, ADR/HG/ZnCl_2_ *versus* ADR/HG, not statistically significant (NSS) ADR/ZnCl_2_ *versus* ADR)

**Figure 5:**
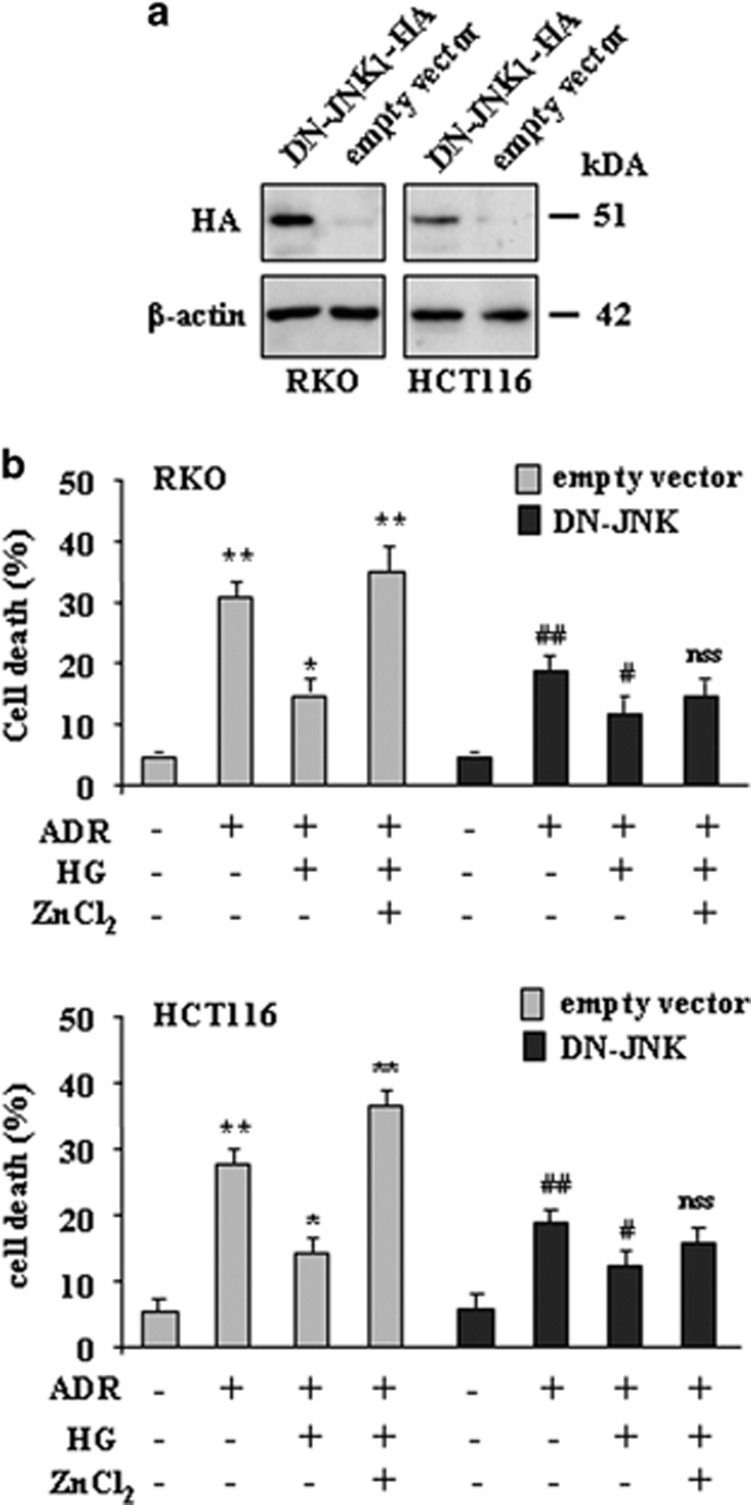
ZnCl_2_ cotreatment does not re-establish the ADR-induced cell death, inhibited by HG, in DN-JNK-HA cells. (**a**) RKO and HCT116 cells were stably transfected with JNK1-APF-HA mutant (dominant negative (DN-JNK-HA)) or with control vector (empty vector) and transfected protein was detected by western immunoblotting with anti-HA antibody. Anti-*β*-actin was used as protein loading control. The predicted molecular weight is indicated (kDa). (**b**) Control cells and cells transfected with DN-JNK-HA were treated with ADR (2 *μ*g/ml) in low and high glucose condition with or without ZnCl_2_ (100 *μ*M) for 24 h, before being assayed for cell viability by Trypan blue exclusion. Data are presented as mean±S.E.M. (*n*=6) (one-way ANOVA plus Bonferroni test, **P<0.001 ADR (empty vector) *versus* Mock (empty vector), ADR/HG/ZnCl_2_ (empty vector) *versus* ADR/HG (empty vector), ^##^*P*<0.001 ADR (DN-JNK) *versus* Mock (DN-JNK), ADR (DN-JNK) *versus* ADR (empty vector), ^#^*P*<0.01 ADR/HG (DN-JNK) *versus* ADR (DN-JNK), *P<0.01 ADR/HG *versus* ADR (DN-JNK), not statistically significant (NSS) ADR/HG/ZnCl_2_ (DN-JNK) *versus* ADR/HG (DN-JNK)

To highlight the apoptotic mechanism triggered by ZnCl_2_ in this HG/ADR setting, we attempted to analyze the JNK pathway as JNK activation has been demonstrated to be required for apoptosis caused by chemotherapeutic agents such as ADR.^19, 41^ As shown in Figure 4, the levels of phosphorylated (p)-JNK were significantly increased after ADR treatment and almost suppressed by HG condition; interestingly, ZnCl_2_ cotreatment counteracted the negative effect of HG and re-established the p-JNK levels in both cell lines (Figure 4). To evaluate whether JNK activation might contribute to apoptosis induction by ADR, RKO and HCT116 cells were stably transfected with the HA-JNK1-APF mutant (DN-JNK-HA),^42^ which functions as a dominant negative of endogenous JNK activity,^43^ or with an empty control vector for comparison (Figure 5a). As shown in Figure 5b, stable overexpression of the inactivatable DN-JNK-HA mutant significantly reduced the ADR-induced cell death (Figure 5b, compare ADR (DN-JNK) with ADR (empty vector)), although it was not abolished, suggesting that other apoptotic pathways were involved independently of JNK activation. Of note, when DN-JNK was overexpressed, HG condition still inhibited the ADR-induced cell death (Figure 5b, compare ADR/HG *versus* ADR (DN-JNK) with ADR/HG *versus* ADR (empty vector)), whereas ZnCl_2_ cotreatment was no longer able to re-establish cell death in ADR/HG cells (Figure 5b, ADR/HG/ZnCl_2_ *versus* ADR/HG (DN-JNK) with ADR/HG/ZnCl_2_ *versus* ADR/HG (empty vector)). This finding suggests that JNK is one of the ZnCl_2_ apoptotic target that can overcome the HG inhibitory effect.

**Figure 6:**
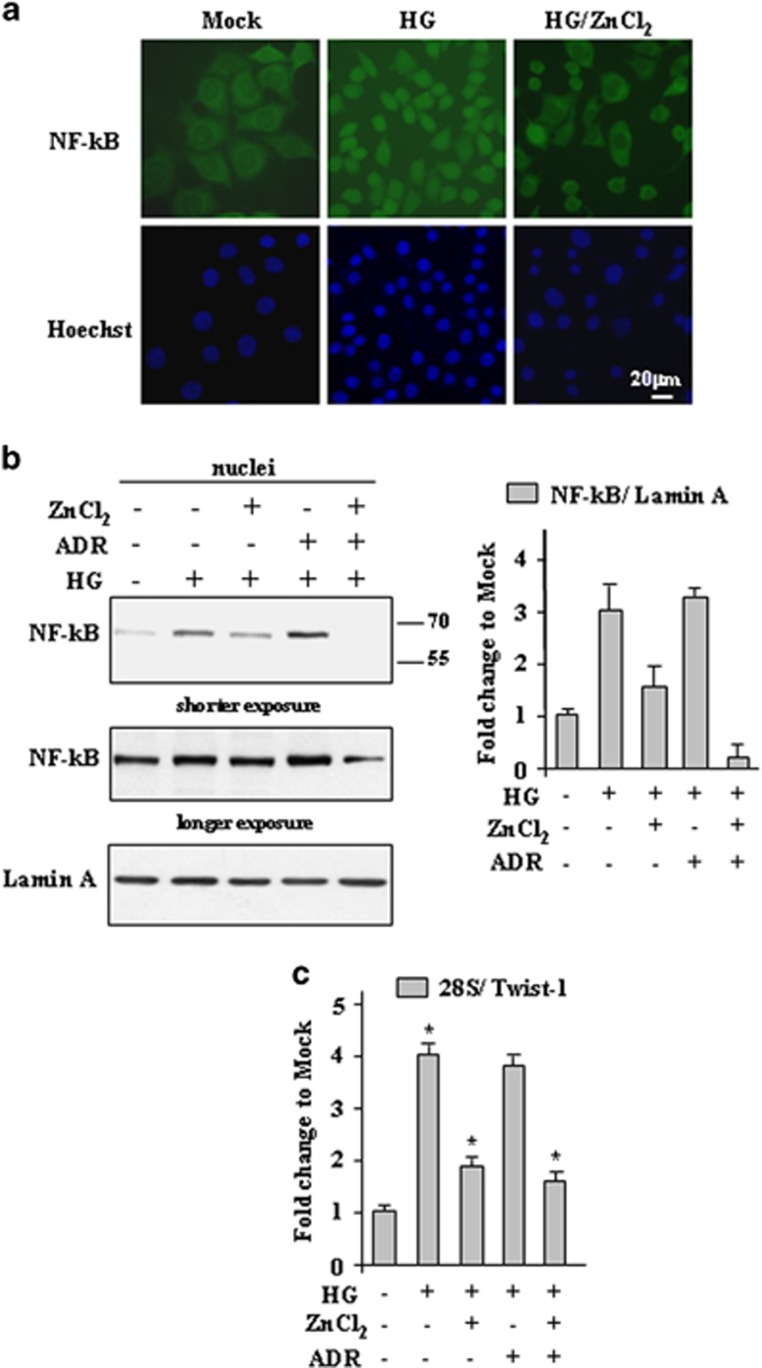
ZnCl_2_ impairs NF-*κ*B nuclear localization in HG condition. (**a**) RKO cells were grown in HG condition for 24 h with or without ZnCl_2_ (100 *μ*M) before being assayed for immunocytochemistry with anti-NF-*κ*B antibody. Nuclei were stained with Hoechst. (**b**) Western blotting and relative quantification of nuclear NF-*κ*B/Lamin A ratio in RKO cells grown in HG condition for 24 h with or without ZnCl_2_ (100 *μ*M) and ADR (2 *μ*g/ml) treatment. Data represent mean±S.D. (**c**) Cells treated as in (**a**) were assayed for semi-quantitative RT-PCR of Twist-1 gene; 28S was used as a control for efficiency of RNA extraction and transcription. Densitometric analysis for quantification of 28S/Twist-1 ratio is presented as mean±S.E.M. of *n*=4 (RT-PCR) (one-way ANOVA plus Bonferroni test, *P<0.01 HG *versus* Mock, HG/ZnCl_2_ *versus* HG, HG/ADR/ZnCl_2_ *versus* HG/ADR)

HG has been shown to increase nuclear translocations of p65, a component of NF-*κ*B transcription factor^30^ that may have antiapoptotic activity by inhibiting JNK.^31, 32^ In the attempt to evaluate the mechanism of JNK activation by ZnCl_2_ in HG condition, we analyzed NF-*κ*B cellular localization and target gene transcription. Immunofluorescent analysis of NF-*κ*B p65 showed that HG condition increased NF-*κ*B nuclear translocation that could be efficiently counteracted by ZnCl_2_ cotreatment that allowed a more diffuse cytoplasmic staining (Figure 6a). In addition, western blot analysis of nuclear extracts showed that HG-induced NF-*κ*B nuclear translocation was not affected by ADR treatment, whereas it was markedly inhibited in the presence of ZnCl_2_ alone or in combination with ADR (Figure 6b), as also assessed by densitometric analysis (Figure 6b, right panel). The NF-*κ*B activation was evaluated by monitoring the expression of its target gene Twist-1.^44^ As shown in Figure 6c, the HG-induced Twist-1 mRNA expression was not affected by ADR treatment, whereas it was markedly inhibited in the presence of ZnCl_2_ alone or in combination with ADR, in agreement with the results of NF-*κ*B nuclear translocation. Altogether, these findings suggest that JNK activation is one of the apoptotic pathways activated by ZnCl_2_ in HG condition and that this activation correlated with inhibition of the antiapoptotic pathway dictated by HG-induced NF-*κ*B.

## Discussion

Hyperglycemia, or high blood glucose, occurs during several pathological processes including diabetes mellitus, obesity, pancreatitis, chronic stress, and cancer.^1, 2, 3, 4, 5, 6, 7^ It appears that hyperglycemia may induce cancer cells to undergo a series of genetic and metabolic changes that allow them to develop a more malignant phenotype but also to become more resistant to conventional antineoplastic therapies including chemical agents.^8, 9, 10, 11, 12^ Therefore, controlling hyperglycemia may have important therapeutic implications for cancer patients. However, the role of hyperglycemia in cancer therapy and the exact mechanisms remain unclear. Few mechanisms have been so far implicated as responsible for chemoresistance when hyperglycemia occurs, such as overexpression of fatty acid synthase (FAS),^8^ glucose-induced upregulation of insulin growth factor binding protein (IGFBP2),^10^ and inhibition of oncosuppressor p53 apoptotic activity.^36, 45^ Here we show that colon cancer cells cultured in HG condition *in vitro* developed resistance to apoptotic cell death induced by the antineoplastic agent ADR; such resistance was in part due to HG-induced upregulation of GLUT1 gene expression and to inhibition of JNK apoptotic pathway likely through NF-*κ*B activation. In the attempt to counteract the molecular changes induced by HG condition, we found that ZnCl_2_ treatment impaired the HG-induced GLUT1 gene expression as well as NF-*κ*B activation and that such molecular changes correlated with restoration of the cytotoxic effect of ADR inhibited by HG.

It has been shown that HG induces modulation of oncogenic pathways,^46^ including activation of HIF-1, leading to increased expression of genes associated with glucose metabolism and transportation, angiogenesis, and survival/antiapoptotic processes.^20^ HIF-1 is a heterodimeric transcription factor that consists of two subunits, HIF-1*α* and HIF-1*β*; HIF-1*β* is constitutively expressed in cells, whereas HIF-1*α* stability is stimulated by hypoxia, growth factors, and several oncogenes.^20^ HIF-1*α* is mostly regulated at posttranscriptional levels by low oxygen conditions;^47^ however, some studies showed that HIF-1*α* can be upregulated at a transcriptional level independently of the oxygen context.^48^ HIF-1*α* has a broad impact on the expression of many genes involved in cell proliferation, motility, and apoptosis, and among them is GLUT1 along with a panel of glycolysis genes.^29^ Glucose is a major source of energy, and increased GLUT1 expression may indicate an increased utilization of energy that in turn may correlate with poor prognosis^25^ and resistance to antineoplastic therapies. The glucose transporter GLUT1 is often overexpressed in several solid tumors and is associated with tumor progression and chemoresistance.^25, 26, 27^ In addition, the expression of GLUT1 often correlates with the ability to detect tumors by PET.^49^ Overexpression of GLUT1 gene has been shown to confer two- to fivefold higher drug resistance in SW620 and K562 to ADR.^50^ However, inhibition of glucose uptake sensitizes cancer cells to ADR-induced cell apoptosis to overcome drug resistance;^50^ similarly, reduction of GLUT1 expression by siRNA leads to a reduction of glucose transport, glucose consumption, and lactate secretion suggestive of reduced glycolysis and glucose metabolism.^51^ In agreement, we found here that HG condition induced GLUT1 gene expression that correlated with reduced ADR cytotoxic effect; interference of GLUT1 expression by siRNA counteracted the effect of HG, improving the ADR-induced cell death. Comparable outcome was obtained by using ZnCl_2_ that reduced the HG-induced GLUT1 overexpression and re-established the ADR cytotoxicity. The mechanism of ZnCl_2_-dependent modulation of such mRNA expression has not been elucidated here; however, GLUT1 as well as HK2 expressions have been shown to be upregulated by HIF-1*α*^28^ and ZnCl_2_ has been shown to downregulate HIF-1*α* expression and inhibit its transcriptional activity,^33, 35^ and hence we can speculate that, in this setting, ZnCl_2_ likely acted on HIF-1 activity to downregulate genes such as GLUT1 and HK2. The final biological outcome of ZnCl_2_ treatment was similar to that obtained by the glycolytic inhibitor 2-DG whose association with chemotherapeutic agents has been successfully used in preclinical and clinical trials.^38, 39^ Therefore, targeting GLUT1 or the glycolytic pathway by ZnCl_2_ might be considered a useful strategy to reverse the drug resistance in HG condition, to be exploited in the future. This is also supported by studies showing the use of zinc as a potential coadjuvant for type 2 diabetes because of its beneficial effects on glycemic control.^52^

Another mechanism by which HG could increase chemoresistance is both the inhibition of ADR-induced JNK phosphorylation and the activation of NF-*κ*B transcription factor. JNK is an apoptotic signaling pathway known to be activated by ADR.^18, 19^ On the other hand, activation of NF-*κ*B transcription factor may be elicited by hyperglycemia.^30^ NF-*κ*B activation may have a double-edged role in cancer. On one hand, activation of NF-*κ*B is part of the immune defense that targets and eliminates transformed cells, and on the other hand, NF-*κ*B can exert a variety of protumorigenic functions in many types of cancer.^31^ Among them, the NF-*κ*B antiapoptotic effect may depend by both blocking the activation of JNK^32^ and by inducing Twist-1 target gene, a new mediator of the protective activity of NF-*κ*B.^44^ Notably, this protective activity of Twist-1 is capable of blocking both the apoptotic and the necrotic pathways activated by chemotherapeutic agents, thus inducing chemoresistance.^44^ In agreement, we found here that HG-induced NF-*κ*B activation, as assessed by its nuclear translocation and transcription of Twist-1, could be inhibited by ZnCl_2_. This finding is in agreement with a previous study showing that zinc targets NF-*κ*B activity.^53^ Therefore, blocking NF-*κ*B/Twist-1 antiapoptotic pathway correlated with increased cancer cell response to ADR cytotoxicity in HG condition. This effect could likely depend on restoration of JNK pathway, although the mechanistic contribution of those pathways needs to be further clarified in this setting.

In summary, we show here that a combination treatment with ADR and ZnCl_2_ re-established the ADR cytotoxic effect inhibited by HG condition, through modulation of several, partially interconnected, apoptotic/chemoresistant pathways. Zinc supplementation has recently attracted researchers for improvement of anticancer chemotherapeutic therapies. Zinc, a trace element, is essential for the wide range of physiological processes, including growth, development, and immune functions as well as the intracellular activities of ∼300 enzymes and 2000 transcription factors and has been shown to play a role in cancer prevention.^54^ Thus, ZnCl_2_ or zinc compounds have been used in *in vitro* and *in vivo* studies to improve the cytotoxic effect of antineoplastic agents through several mechanisms.^55, 56, 57, 58, 59, 60, 61, 62^ If experimentally verified in *in vivo* model(s) of hyperglycemia, such combination therapy with ZnCl_2_ and antineoplastic agents may lead to novel means toward management of hyperglycemic cancer patients.

## Materials and Methods

### Cell culture and reagents

In this study colon cancer RKO and HCT116 cells were used. Cells were routinely cultured in DMEM (Life Technology-Invitrogen) containing 1 g/l D-glucose (low glucose), supplemented with 10% heat-inactivated fetal bovine serum (FBS) plus 100 units/ml penicillin/streptomycin and glutamine in 5% CO_2_ humidified incubator at 37 °C. For HG treatment, low glucose culture medium was replaced with DMEM containing 4.5 g/l D-glucose (Life Technology-Invitrogen, Carlsbad, CA, USA) supplemented with 2% FBS for 24 h, as previously reported.^10, 12, 36^ Chemotherapeutic drug ADR and ZnCl_2_ were added in culture medium at 2 *μ*g/ml and 100 *μ*M,^36^ respectively, for the indicated times. Glycolytic inhibitor 2-DG^34^ (Santa Cruz Biotechnology, Dallas, TX, USA) was dissolved in DMSO and added to culture medium at 12 mM concentrations for 24 h. Irreversible, cell-permeable, pancaspase inhibitor zVAD-fmk^63^ (Calbiochem, San Diego, CA, USA) was diluted in DMSO, stored at −20 °C and used at a final concentration of 40 *μ*M for 16 h.

### Viability assay

For viability assay, subconfluent cells were plated in duplicate in 60 mm multiwell Petri dishes and 24 h later culture medium was replaced with HG or low-glucose medium, both containing 2% FBS. The day after, ADR (2 *μ*g/ml) and ZnCl_2_ (100 *μ*M) were added to cell cultures for 24 h. Both floating and adherent cells were collected and cell viability was determined by Trypan blue exclusion by direct counting with a hemocytometer, as previously reported.^36^ The percentage of cell death, as blue/total cells, was assayed by scoring ∼200 cells per well in triplicate. Bright-field images were taken in a Nikon Eclipse TS100 microscope equipped with a Nikon ELWD camera (Nikon Instruments Europe BV, Amsterdam, The Netherlands).

### Cell death/PI staining

Cell death was quantified by FACS analysis, staining cells with the nonvital dye PI (Immunological Sciences, Rome, Italy), following the manufacturer's instruction. Briefly, cells floating were collected by centrifugation and pooled with adherent cells recovered from the plates, fixed in 80% ethanol, and stained in a PBS solution containing PI (62.5 mg/ml; Sigma-Aldrich, St. Louis, MO, USA) and RNase A (1.125 mg/ml; Sigma-Aldrich). Samples were acquired with a FACScan instrument (Becton Dickinson Europe Holdings SAS, Le Pont De Claix, France) and the percentage of cells in sub-G1 compartment was calculated using ModFit LT software (Becton Dickinson). Approximately 30 000 events were acquired and gated using forward scatter and side scatter to exclude cell debris.

### Western blot analysis

Total cell extracts were prepared by incubation in lysis buffer (50 mM Tris-HCl, pH 7.5, 150 mM NaCl, 5 mM EDTA, pH 8.0, 150 mM KCl, 1 mM dithiothreitol, 1% Nonidet P-40) and a mix of protease and phosphatase inhibitors (Sigma-Aldrich Chemical Company, Sigma-Aldrich). Nuclear extracts were prepared essentially as previously described.^61^ Briefly, cells were suspended in hypotonic buffer (10 mM HEPES, pH 7.9, 10 mM KCl, 0.1 mM EDTA, 0.1 mM EGTA) and placed on ice for 15 min. Nonidet P-40 was added to a final concentration of 0.5%. Cells were spun top speed for 30 s and the supernatant (cytoplasmic fraction) was discharged. The remaining pellet was washed with hypotonic buffer, resuspended in lysis buffer as above, and spun at 15 000 × *g* for 15 min to remove debris and collect the supernatant (nuclear fraction). Protein concentration was then determined using BCA Protein Assay kit (Bio-Rad, Hercules, CA, USA). Samples were then denatured in SDS sample buffer. Total or nuclear cell lysates (20–60 *μ*g protein/lane) were resolved by 9–18% SDS-polyacrylamide gel electrophoresis and transferred to polyvinylidene difluoride (PVDF) membranes (Merck Millipore, Billerica, MA, USA). Unspecific binding sites were blocked by incubating membranes for 1 h in 0.05% Tween-20 (v/v in TBS) supplemented with 5% non-fat powdered milk or bovine serum albumin, followed by overnight incubation with the following primary antibodies: rabbit polyclonal cyclin B1, rabbit polyclonal p65 NF-*κ*B, rabbit polyclonal Lamin A (Santa Cruz Biotechnology, Santa Cruz, CA, USA), mouse monoclonal anti-PARP (cleavage site-214/215, Millipore, Billerica, MA, USA), rat monoclonal HA (Roche S.p.A, Milan, Italy), rabbit polyclonal p-JNK, and total JNK (Cell Signaling Technologies, Danvers, MA, USA). Equal lane loading was monitored by probing membranes with antibodies specific for mouse monoclonal *β*-actin (Calbiochem, San Diego, CA, USA). Primary antibodies were detected with appropriate anti-immunoglobulin-G-horseradish peroxidase secondary antibodies (Bio-Rad). Enzymatic signals were visualized using chemiluminescence (ECL Detection system, Amersham GE Healthcare, Milan, Italy).

### RNA extraction and semiquantitative RT-PCR analysis

Cells were harvested in TRIzol Reagent and total RNA was isolated following the manufacturer's instructions (Invitrogen, Carlsbad, CA, USA). The first-strand cDNA was synthesized from 2 *μ*g of total RNA with MuLV reverse transcriptase kit (Applied Biosystems, Foster City, CA, USA). Semi-quantitative RT-PCR was carried out using Hot-Master Taq polymerase (Eppendorf, Milan, Italy) with 2 *μ*l cDNA reaction and gene-specific oligonucleotides under conditions of linear amplification. PCR products were run on 2% agarose gels and visualized with ethidium bromide. The housekeeping 28S gene, used as internal standard, was amplified from the same cDNA reaction mixture. Densitometric analysis was applied to quantify mRNA levels compared with control gene expression.

### Transfection and plasmids

Cells were transfected using the LipofectaminePlus method (Invitrogen) according to the manufacturer's specifications. For stable transfection, 4 × 10^5^ RKO and HCT166 cells were transfected with the nonphosphorylatable (competitive inhibitor) HA-JNK1-APF (JNK dominant negative(DN-JNK-HA)) expression vector^42^ (kindly provided by Lynn E Heasley, University of Colorado, Aurora, CO, USA). At 48 h after transfection, cells underwent selection with geneticin G418 (1 mg/ml). G418-resistant cells were pooled as mixed population 2 weeks later and used throughout the paper.

### Immunocytochemistry

Cells were cultured to appropriate density in 35 mm culture dishes or 6-well plates. The day after plating cells were treated with HG and HG/ZnCl_2_ were applied for 24 h. After treatment, culture medium was removed and cells were fixed with 10% formalin for 30 min at room temperature and then with ice-cold methanol for 5–10 min. To block nonspecific antibody binding, cells were incubated in 10% normal goat serum from 1 h to overnight at room temperature. Primary antibody NF-*κ*B p65 (Santa Cruz Biotechnology) was added in a 1 : 50 dilution and incubated at room temperature from 2 h to overnight at room temperature and then with secondary antibody for 1 h at room temperature. Nuclei were then stained with Hoechst 33258 for 10 min and cover-slipped using 30% glycerol, with appropriate sealing. Cells were then visualized on a Nikon Eclipse Ti-U fluorescence microscope (Nikon).

### siRNA interference

Cells were plated at semiconfluence in 35-mm Petri dishes the day before transfection. Control-siRNA and siGLUT1 (Dharmacon, Thermo-Scientific, Fisher Scientific SAS, Illkirch Cedex, France) were transfected overnight using LipofectaminePlus reagent (Invitrogen). GLUT1 silencing was evaluated 48 h after transfection by RT-PCR analysis.

### Statistical analysis

Each experiment, unless specified, was performed at least three times. Results are expressed as values of mean±S.D. Statistical significance was determined using Student's *t*-tests for two sample comparisons and one-way ANOVA analysis for three or more sample comparisons.

## Abbreviations

2-DG: 2-deoxy-D-glucose
ADR: adryamicin
FACS: fluorescence-activated cell sorting
DN-JNK1: JNK1-APF mutant
GLUT1: glucose trasporter isoform 1
HG: high glucose
HIF-1*α*: hypoxia-inducible factor-1*α*
HK2: enzyme hexokinase 2
JNK/SAPK: c-Jun N-terminal kinase/stress-activated protein kinase
NF-*κ*B: nuclear factor *κ*-light-chain-enhancer of activated B cells
PARP: poly(ADP-ribose) polymerase
PI: propidium iodide
PVDF: polyvinylidene difluoride
RT-PCR: reverse transcription-PCR
siRNA: small interference RNA
ZnCl_2_: zinc chloride
